# Impact of the heavy-quark matching scales in PDF fits

**DOI:** 10.1140/epjc/s10052-017-5407-3

**Published:** 2017-12-07

**Authors:** The xFitter Developers’ Team:, V. Bertone, D. Britzger, S. Camarda, A. Cooper-Sarkar, A. Geiser, F. Giuli, A. Glazov, E. Godat, A. Kusina, A. Luszczak, F. Lyonnet, F. Olness, R. Plačakytė, V. Radescu, I. Schienbein, O. Zenaiev

**Affiliations:** 1 Department of Physics and Astronomy, VU University, 1081 HV Amsterdam, The Netherlands; 2 Nikhef Theory Group Science Park 105, 1098 XG Amsterdam, The Netherlands; 3 DESY Hamburg, Notkestraße 85, 22609 Hamburg, Germany; 4 CERN, 1211 Geneva 23, Switzerland; 5 University of Oxford, 1 Keble Road, Oxford, OX1 3NP UK; 6 SMU Physics, Box 0175, Dallas, TX 75275-0175 USA; 7 Laboratoire de Physique Subatomique et de Cosmologie, Université Grenoble Alpes, CNRS/IN2P3, 53 avenue des Martyrs, 38026 Grenoble, France; 8 Institute of Nuclear Physics, Polish Academy of Sciences, ul. Radzikowskiego 152, 31-342 Kraków, Poland; 9 T.Kosciuszko Cracow University of Technology, 30-084 Kraków, Poland; 10 Institut für Theoretische Physik, Universität Hamburg, Luruper Chaussee 149, 22761 Hamburg, Germany

## Abstract

We investigate the impact of displaced heavy-quark matching scales in a global fit. The heavy-quark matching scale $\mu _{m}$ determines at which energy scale $\mu$ the QCD theory transitions from $N_{F}$ to $N_{F}+1$ in the variable flavor number scheme (VFNS) for the evolution of the parton distribution functions (PDFs) and strong coupling $\alpha _S(\mu )$. We study the variation of the matching scales, and their impact on a global PDF fit of the combined HERA data. As the choice of the matching scale $\mu _{m}$ effectively is a choice of scheme, this represents a theoretical uncertainty; ideally, we would like to see minimal dependence on this parameter. For the transition across the charm quark (from $N_{F}=3$ to 4), we find a large $\mu _m=\mu _{c}$ dependence of the global fit $\chi ^2$ at NLO, but this is significantly reduced at NNLO. For the transition across the bottom quark (from $N_{F}=4$ to 5), we have a reduced $\mu _{m}=\mu _b$ dependence of the $\chi ^2$ at both NLO and NNLO as compared to the charm. This feature is now implemented in xFitter 2.0.0, an open source QCD fit framework.

## Introduction

The global analysis of PDFs has progressed significantly in recent years. On the experimental front, there is data ranging from the fixed-target regime at low energy, on to HERA and the LHC at very high energies. On the theoretical front, the analysis can be performed not only at NLO, but now at NNLO. To capitalize on these advances, it is essential to include a proper treatment of the heavy quarks to enable high-precision phenomenological analysis of measurements.

The variable flavor number scheme (VFNS) allows us to deal with the heavy-quark mass scale across the full kinematic range by varying the number of active flavors ($N_F$) in the DGLAP QCD evolution [1–11]. At low energy scales, the DGLAP evolution only involves $N_F$ light flavors, and there is no PDF for the heavy quark. At high energy, the heavy-quark PDF is included in the DGLAP evolution so that there are now $N_F+1$ active flavors. To combine the above $N_F$ and $N_F+1$ sub-schemes into a single VFNS, we must define an energy scale $\mu _m$ where we match these together; this will be the scale where we introduce the heavy-quark PDF.

Historically, the matching scale $\mu _m$ was taken to be the heavy-quark mass $m_H$. At the matching scale, the PDFs and $\alpha _S(\mu )$ for $N_F+1$ are defined in terms of the $N_F$ quantities by the following boundary conditions:

1 $$\begin{aligned}&f_i^{(N_F+1)}(x,\mu _m)\nonumber \\&\quad = \sum _{j} \ \mathcal{M}_{i}^{j} \ \otimes \ f_j^{(N_F)}(x,\mu _m) \end{aligned}$$

2 $$\begin{aligned}&\alpha _S^{(N_F+1)}(\mu _m)= \alpha _S^{(N_F)}(\mu _m)\nonumber \\&\quad \times \ \left( 1+ \sum _{n=1}^{\infty } \sum _{k=0}^{n} c_{n \, k} \left[ \alpha _S^{(N_F)}(\mu _m) \right] ^n \ \ln ^k \frac{\mu _m^2}{m_H^2} \right) . \end{aligned}$$

The matching matrix $\mathcal{M}_{i}^{j}$ and coefficients $c_{n \, k}$ can be perturbatively computed.^1^

The new xFitter 2.0.0 program^2^ links to the APFEL code [18] which has implemented generalized matching conditions that enable the switch from $N_F$ to $N_F+1$ at an arbitrary matching scale $\mu _m$. This allows us to introduce the heavy-quark PDF at any scale—not just at $\mu _m=m_H$; this flexibility provides a number of advantages. For example, as the matching scale moves to higher scales, the theory at the lower scales effectively becomes a fixed flavor number scheme (FFNS); yet we still retain a VFNS at the higher scales.

The choice of the matching scale $\mu _m$, like the choice of VFNS or FFNS, amounts to a theoretical scheme choice. As such, the variation of $\mu _m$ represents a source of theoretical uncertainty. The variable matching scale implemented in xFitter provides a new incisive tool to study the impact of these choices across a broad kinematic region. Additionally, as we move from NLO to NNLO calculations, new features are encountered, and these compel us to reexamine some of the foundational elements used to construct this theoretical framework.

Reconsidering the historical choice $\mu _m = m_H$ is of particular relevance for heavy-quark initiated processes at the LHC. In this context, the benefits of the FFNS close to the threshold region and of the VFNS at higher scales are often simultaneously needed to describe the data. Therefore, a careful choice of the matching scales could help formulate a matching prescription between FFNS and VFNS able to achieve this goal in a very simple fashion [19].

This study will examine the combined HERA data set and evaluate the impact of the matching scale on the features of the fit of PDFs. In Sect. 2, we review the key elements of the VFNS used in this study. Section 3, shows the impact of the matching scale $\mu _m$ on the PDFs. In Sect. 4, we perform a fit of the combined HERA data sets at both NLO and NNLO, and investigate the effect of the matching scale $\mu _m$. Section 5 presents an example of how the $\mu _m$ flexibility can be used as a tool to evaluate a recent suggestion for a $N_F$ dependent PDF. Section 6 summarizes the general characteristics and conclusions of this study.

## Variable flavor number scheme (VFNS)

Here we will outline the key concepts of the heavy-quark VFNS which are relevant for this investigation.

### The matching scale $\mu _{m}$

A generalized formulation of the VFNS factorization is based on the Collins–Wilczek–Zee (CWZ) renormalization scheme which involves a sequence of sub-schemes parameterized by the number of active quark flavors ($N_{F}$) [20, 21]. For each sub-scheme, the $N_F$ (active) flavors are renormalized using the $\overline{\text{ MS }}$ scheme while the heavy (inactive) flavors are renormalized using zero-momentum subtraction. This ensures that to all orders in perturbation theory (i) the results are gauge invariant, (ii) the results for the active $N_F$ flavors match the standard $\overline{\text{ MS }}$ results, and (iii) the heavy (inactive) flavors manifestly decouple.^3^ Specifically, both the DGLAP evolution kernels for the $N_F$ active PDFs and the renormalization group equation for $\alpha _S^{(N_F)}(\mu )$ are pure $\overline{\text{ MS }}$.

**Fig. 1:**
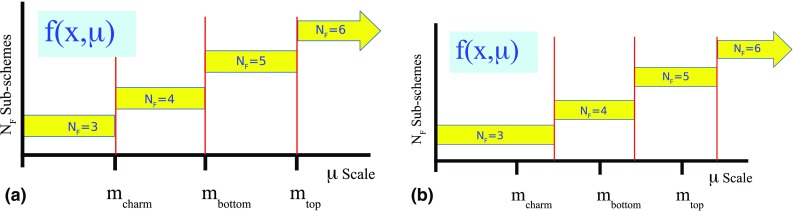
An illustration of the separate $N_{F}$ renormalization sub-schemes which define a VFNS. Historically, the matching scales $\mu _{m}$ were chosen to be exactly the mass values $m_{c,b,t}$ as in **a**. **b** Generalized case where the matching scales $\mu _{m}$ are chosen to be different from the mass values

To connect the separate $N_F$ sub-schemes into a single scheme that spans the full kinematic range, we must choose a matching scale $\mu _{m}$ which will relate the sub-schemes. This is where we define the PDFs and $\alpha _S$ of the $N_F+1$ scheme in terms of the $N_F$ scheme, cf. Eqs. (1) and (2). A schematic representation of this is displayed in Fig. 1.

For example, at scales $\mu _c< \mu <\mu _b$ the scheme has $N_F=4$ active flavors $\{u,d,s,c\}$ with 4-flavor PDFs and $\alpha _S^{(4)}(\mu )$; the bottom quark is *not* treated as a parton and $f_b^{(4)}(x,\mu )=0$.

At the scale $\mu =\mu _b$, we can compute the 5-flavor PDFs and $\alpha _S^{(5)}(\mu )$ in terms of the 4-flavor quantities; the boundary conditions are non-trivial and the PDFs and $\alpha _S(\mu )$ are not necessarily continuous. This scheme has $N_F=5$ active flavors $\{u,d,s,c,b\}$, and the bottom quark is included in the DGLAP evolution.

### Historical choice of $\mu _{m}=m_{c,b,t}$

Historically, the matching scale $\mu _{m}$ was commonly taken to be exactly equal to the mass of the heavy quark, $\mu _{m}=m_{c,b,t}$; this was a convenient choice for a number of reasons.

For example, the generic NLO matching condition for the PDFs at the $N_F=4$ to $N_F=5$ transition is [22]:

3 $$\begin{aligned}&f_{i}^{(5)}(x,\mu _b) \nonumber \\&\quad =\left\{ \delta _{ij} + \frac{\alpha _{S}^{(4)}(\mu _b)}{2\pi } \left[ c_{0}^{ij}+c_{1}^{ij} \ln \left( \frac{\mu _b^2}{m_b^2} \right) \right] \right\} \otimes f_{j}^{(4)}(x,\mu _b) \nonumber \\ \end{aligned}$$

where $c_{0}^{ij}$ and $c_{1}^{ij}$ are perturbatively calculable coefficient functions. Note that the right-hand side uses 4-flavor PDFs and $\alpha _S$, while the left-hand side uses 5-flavors.

The choice $\mu _{b}=m_{b}$ will cause the logarithms to vanish, and this greatly simplifies the matching relations. Additionally, at NLO in the $\overline{\text{ MS }}$ scheme the constant term $c_{0}^{ij}$ in the matching equation coincidentally vanishes [14]. The net result is that, for $\mu _{b}=m_{b}$, the PDFs will be continuous (but not differentiable) at NLO. This is historically why $\mu _m$ was set to $m_{c,b,t}$.

However, at NNLO and beyond the situation is more complex; in particular, the higher-order terms corresponding to $c_{0}^{ij}$ will be non-zero, and the matching of both the PDFs and $\alpha _S(\mu )$ will be discontinuous. Consequently, the freedom to arbitrarily choose the matching scale $\mu _{m}$ (and decide where to place the discontinuities) will have a number of advantages, as the next subsection will demonstrate.

### Smooth matching across flavor thresholds 

To gauge the impact of the contributions of the heavy-quark PDFs in a process independent manner, we can compare the DGLAP evolved heavy-quark PDF $f_b(x,\mu )$ with a perturbatively computed quantity: $\widetilde{f}_b(x,\mu )$. At NLO, $\widetilde{f}_b(x,\mu )$ takes a gluon PDF and convolutes it with a perturbative (DGLAP) splitting $g\rightarrow b \bar{b}$ [23, 24]; this can be thought of as a “perturbatively” computed bottom PDF. The result at NLO is

4 $$\begin{aligned} \widetilde{f}_b(x,\mu ) = \frac{\alpha _S}{2 \pi }\ P_{g\rightarrow b \bar{b}}\otimes f_g \ \ \ln \left[ \frac{\mu ^2}{m_b^2} \right] . \end{aligned}$$

The difference between $f_b(x,\mu )$ and $\widetilde{f}_b(x,\mu )$ is due to the higher-order terms which are resummed by the heavy-quark DGLAP evolution.^4^

To better understand these quantities, we compute DIS bottom production at NLO in a 5-flavor VFNS, and find the cross section to be [3]:

5 $$\begin{aligned} \sigma _{\scriptscriptstyle {\mathrm{VFNS}}}= \sigma _{b\rightarrow b}\otimes \left[ f_b(x,\mu ) - \widetilde{f}_b(x,\mu ) \right] + \underbrace{ \sigma _{g\rightarrow b}\otimes f_g(x,\mu ) }_{\displaystyle {\sim \sigma _{\scriptscriptstyle {\mathrm{FFNS}}}}}. \end{aligned}$$

Here, $\sigma _{b\rightarrow b} \otimes f_b$ is the LO term, and $\sigma _{b\rightarrow b} \otimes \widetilde{f}_b$ is the subtraction (SUB) term. The unsubtracted NLO term $\sigma _{g\rightarrow b}\otimes f_g$ corresponds (approximately) to a FFNS calculation. Here, $\sigma _{b \rightarrow b}$ is proportional to a delta function which makes the convolution trivial.

**Fig. 2:**
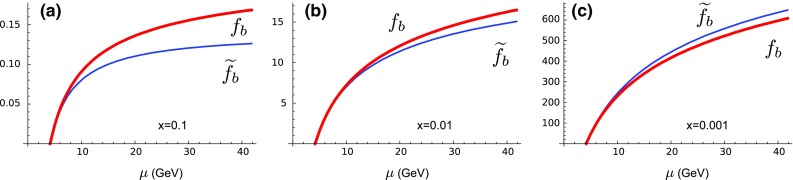
The comparison of the DGLAP evolved PDF $f_b(x,\mu )$ and the perturbatively calculated $\widetilde{f}_b(x,\mu )$ as a function of $\mu$ for selected *x* values. For $\mu \rightarrow m_b$ we find the functions match precisely: $\widetilde{f}_b(x,\mu ) \rightarrow f_b(x,\mu )$. We have used NNPDF30_lo_as_118_nf_6 as the base PDF set

Thus, the combination $(f_b - \widetilde{f}_b)$ represents (approximately) the difference between a VFNS and FFNS result.^5^ These quantities are displayed in Fig. 2. In the region $\mu \sim m_b$, $f_b(x,\mu )$ and $\widetilde{f}_b(x,\mu )$ match precisely; it is this cancellation which (at NLO) ensures physical quantities will have a smooth transition across the flavor threshold.

At larger $\mu$ scales, $f_b(x,\mu )$ and $\widetilde{f}_b(x,\mu )$ begin to diverge; this indicates that the resummed heavy-quark logarithms are becoming sizable. The details clearly depend on the specific *x* values. For large *x* ($x\sim 0.1$) we find $f_b(x,\mu ) >\widetilde{f}_b(x,\mu )$, while for small *x* ($x\sim 0.001$) the result is $f_b(x,\mu ) <\widetilde{f}_b(x,\mu )$; finally, for intermediate *x* ($x\sim 0.01$) the two terms nearly balance even for sizable $\mu$ scales.

While the QCD theory ensures proper matching, this is not so easy to implement in a general numeric calculation for all observables, especially for complex observables involving multiple numeric integrations. In particular, the cancellation of Fig. 2 requires that the quark masses $m_{c,b,t}$, the strong coupling $\alpha _{S}$, and the order of the PDF evolution are exactly matched in (i) the DGLAP evolution that generates the PDFs, (ii) the partonic cross sections that are convoluted with the PDFs, and (iii) the fragmentation function (if used).

In practice, there are almost always slight differences. A typical analysis might use a variety of PDFs from different PDF groups, together with a selection of fragmentation functions; each of these will be generated with a specific set of quark masses and $\alpha _S$ values which are most likely different. Thus, it is essentially inevitable that the cancellations exhibited in Fig. 2 will be spoiled leading to spurious contributions which can be substantive.

Instead of setting the matching scale at the heavy-quark mass $\mu _m=m_{c,b,t}$, xFitter provides the flexibility to delay the matching scale $\mu _{m}$ to a few multiples of the heavy-quark mass; this will avoid the need for the delicate cancellation in the $\mu _m\sim m_{c,b,t}$ region, and the results will be numerically more stable.

As an extreme example, one could imagine delaying the matching scale to infinity ($\mu _{m}\rightarrow \infty$) which would amount to a FFNS; here, the disadvantage is that the FFNS does not include the resummation of the higher-order heavy-quark logs which have been demonstrated to improve the fit to the data [25]. Using the new flexibility of the xFitter program, it is possible to investigate the trade-offs between a large and small value for the matching scale $\mu _{m}$.

A separate example is present in the transverse momentum ($p_T$) distributions for heavy-quark production ($pp\rightarrow b \bar{b}$) using the (general mass) GM-VFNS [26, 27]. If we compute this in an $N_F=5$ flavor scheme, the contribution from the $b\bar{b}\rightarrow b\bar{b}$ sub-process with an exchanged *t*-channel gluon will be singular at $p_T=0$. For a scale choice of the transverse mass $\mu =\sqrt{p_T^2+m_b^2}$ (a common choice), the singularity can be cured by either a different scale choice, or by delaying the switch to the 5-flavor scheme to a higher scale, e.g., $\mu _b\sim 2 m_b$.

### Discontinuities

At NNLO both the PDFs and $\alpha _{S}(\mu )$ will necessarily have discontinuities when matching between the $N_{F}$ to $N_{F}+1$ flavor schemes as specified by Eqs. (1) and (2). If we are analyzing a high-precision experiment and arbitrarily impose a matching at the quark masses $\mu _{m}=m_{c,b,t}$, this may well introduce discontinuities within the kinematic range of some precision data. While it is true that these discontinuities simply reflect the theoretical uncertainties, it is disconcerting to insert them in the middle of a precision data set.

**Fig. 3:**
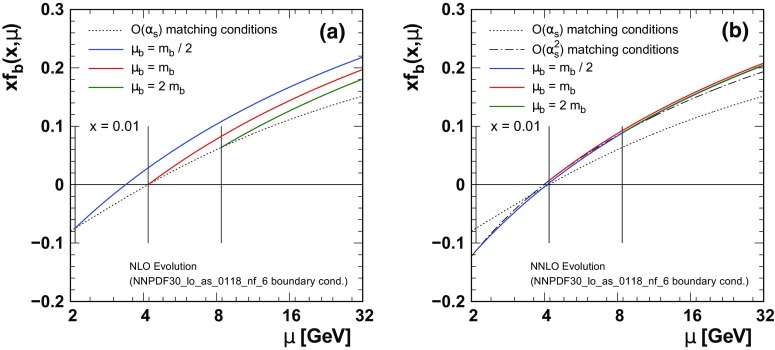
We display the b-quark PDF $x\, f_b^{(5)}(x,\mu )$ for different choices of the matching scales $\mu _{m}=\{m_b/2,m_b,2m_b \}$ (indicated by the vertical lines) computed at NLO (**a**) and NNLO (**b**)

The ability to vary the matching scale $\mu _{m}$ provides us with the option to shift the location of these discontinuities for a particular analysis. For example, to analyze the high-precision charm production HERA data, we necessarily are working in the region of the bottom mass scale ($\sim 4.5$ GeV). Both the PDFs and $\alpha _{S}(\mu )$ will be discontinuous at the matching scale which transitions between the $N_{F}=4$ and $N_{F}=5$ schemes. If the matching scale is chosen in the region $\mu _{m}\sim m_{b}$, these discontinuity will appear in the region of the data. Instead, we can shift the matching $\mu _{m}$ to a higher scale (for example, set $\mu _{m}$ to $2m_{b}$ or $3m_{b}$) and thus analyze the charm production data in a consistent $N_{F}=4$ flavor framework. Yet, we still retain the transition to $N_{F}=5$ flavors so that processes such as LHC data at high scales are computed including the bottom PDF.

## The matching scale $\mu _m$

Having sketched the characteristics of a flexible matching scale $\mu _{m}$, we will examine the specific boundary condition and the impact on the global fit of the PDFs.

### Impact of matching on the PDFs

Figure 3 displays the effect of different values of the bottom matching scale $\mu _{b}$ on the bottom-quark PDF for both the NLO and the NNLO cases.^6^ At NLO, the matching conditions are schematically:^7^

6 $$\begin{aligned} f_{b}^{(5)}(x,\mu _b)= \frac{\alpha _{S}^{(4)}(\mu _b)}{2\pi } \ \left[ c_{0}^{bg}+c_{1}^{bg}\ L\right] \otimes f_{g}^{(4)}(x,\mu _b) \end{aligned}$$

where $L=\ln (\mu _b^{2}/m_{b}^{2})$. The superscripts $\{4,5\}$ identify the number of active flavors $N_{F}$. The gluon and the light quarks also have matching conditions analogous to Eq. (6).

**Fig. 4:**
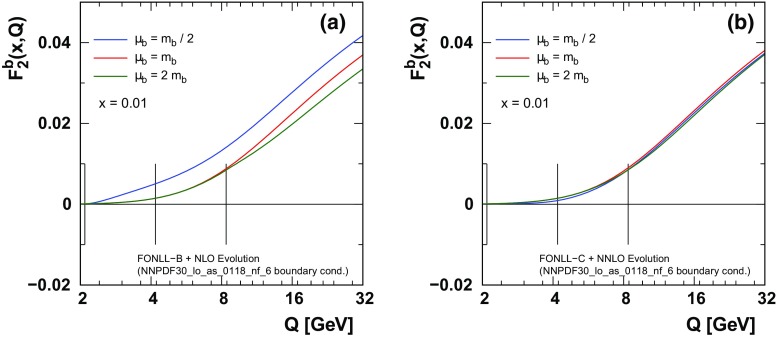
We display$F_2^b(x,Q)$ for different choices of the matching scales $\mu _{m}=\{m_b/2,m_b,2m_b \}$ (indicated by the vertical lines) computed at NLO (**a**) and NNLO (**b**). Here, we have chosen $\mu =Q$. For details of the FONNL calculation see Ref. [6]

As already mentioned, if we choose to match at $\mu _b=m_{b}$ then $L=0$ and $f_{b}^{(5)}(x,\mu _m)$ will start from zero at $\mu _b=m_{b}$. This coincidental zero ($c_{0}^{ij}=0$) is the historic reason why most NLO analyses perform the matching at $\mu _b=m_{b}$; if both the $c_{0}^{ij}$ and the $c_{1}^{ij}\, L$ terms can be ignored, then the PDFs are continuous (but not differentiable) across the matching scale.^8^

At NNLO this is no longer the case; the NNLO constant term at $\mathcal{O}(\alpha _{S}^{2})$ does not vanish and the PDFs will have a discontinuity regardless of the choice of matching scale. Although the difference is subtle, the (red) curve for $\mu _{b}=m_{b}$ does start exactly from zero for the NLO calculation (Fig. 3a), while for the NNLO calculation (Fig. 3b) it starts from a small non-zero value.

As we vary the matching $\mu _{b}$ in the vicinity of $m_{b}$, the sign of $f_{b}^{(5)}(x,\mu _b)$ is controlled by the log term $(c_{1}^{ij}\,L)$. For $\mu _b<m_{b}$ this combination will render $f_{b}^{(5)}(x,\mu _b)$ negative, and this will be compensated (in the sum rule for example) by a positive shift in the 5-flavor gluon. Thus, QCD ensures that both momentum and number sum rules are satisfied to the appropriate order.

Comparing different $f_{b}^{(5)}(x,\mu )$ curves computed with the NLO matching conditions (Fig. 3a) at large $\mu$ scales, there are obvious differences in the curves. This reflects the difference between the single log contribution $(c_{1}^{ij}\, L)$ computed by the matching condition of Eq. (6) and the resummed contributions computed by the DGLAP evolution equation. Specifically, the NLO matching includes the $\alpha _{S}L$ contribution, but it is missing $\alpha _{S}^{2}L^{2}$ and higher terms; this is what gives rise to the differences of Fig. 3a. Obviously, the $\alpha _{S}^{2}L^{2}$ contributions can be important.

Comparing the different $f_{b}^{(5)}(x,\mu )$ curves computed with the NNLO matching conditions (Fig. 3b) at large $\mu$ scales, the differences in the curves are greatly reduced compared to the NLO case. The NNLO result includes both the $\alpha _{S}L$ and the $\alpha _{S}^{2}L^{2}$ contributions, but it is missing $\alpha _{S}^{3}L^{3}$ and higher orders. Clearly the inclusion of the $\alpha _{S}^{2}L^{2}$ contributions dramatically reduces the effect of the different choices of the $\mu _{m}$ matching scale.

Finally, we wish to emphasize that ultimately the choice of $\mu _{m}$ amounts to a choice of scheme. In the limit that perturbation theory is computed to all orders, the infinite tower of logarithms resummed by the DGLAP evolution equations (in the $N_{F}+1$-flavor scheme) will be explicitly summed in the matching conditions (in the $N_{F}$-flavor scheme). In a practical sense, while the differences at NLO are substantive, at NNLO the residual differences at large $\mu$ scale are much smaller. This reduced sensitivity on the choice of $\mu _{m}$ provides increased flexibility and precision in our fits, as will be illustrated in the following sections.

### Impact of matching on $F_2^b(x,Q)$

Having examined the PDFs in the previous section we now turn to a physical observable, $F_2^b(x,Q)$.

Figure 4a shows the NLO result for $F_2^b(x,Q)$ which will receive contributions from the LO process ($\gamma b\rightarrow b$) as well as the NLO ($\gamma g \rightarrow b \bar{b}$) process. For $\mu <\mu _b$, $f_b^{(5)}(x,\mu )=0$ and only the gluon initiated process contributes. For $\mu \gtrsim \mu _b$, the bottom PDF turns on (cf. Fig. 3), and the heavy-quark initiated process now contributes. Because the PDFs, $\alpha _S(\mu )$, and $m_b$ are all carefully matched in this calculation, the cancellation outlined in Sect. 2.3 ensures that the prediction for the physical observable is relatively smooth in this region.

Figure 4b shows the NNLO result for $F_2^b(x,Q)$. As with the PDF matching of Fig. 3b, the additional NNLO contributions significantly reduce the impact of the different matching scales so that the prediction for $F_2^b(x,Q)$ is now very insensitive to $\mu _b$.

**Table 1:** The $\chi ^2$ values at NLO for individual data sets for a selection of the charm matching scales $\mu _c$. The contribution of the charm data contained in the “Correlated $\chi ^2$” and in the “Log penalty $\chi ^2$” terms is indicated separately in the parentheses

Charm NLO	$\mu _c = 1\, m_c$	$2\, m_c$	$3\, m_c$
Charm cross section H1-ZEUS combined [36]	46/47	61/47	54/47
H1 F2 Beauty Vertex [35]	3.1/12	2.8/12	2.7/12
Beauty cross section ZEUS Vertex [37]	12/17	12/17	12/17
HERA1+2 CCep [34]	44/39	44/39	45/39
HERA1+2 CCem [34]	52/42	47/42	48/42
HERA1+2 NCem [34]	220/159	228/159	227/159
HERA1+2 NCep 820 [34]	65/70	70/70	68/70
HERA1+2 NCep 920 [34]	414/377	433/377	471/377
HERA1+2 NCep 460 [34]	221/204	217/204	225/204
HERA1+2 NCep 575 [34]	216/254	224/254	222/254
Correlated $\chi ^2$ total (charm)	86 (10.5)	91 (12.5)	105 (11.3)
Log penalty $\chi ^2$ total (charm)	$+\,6.7\ (+\,0.1)$	$-\,0.7\ (-\,0.4)$	$-\,1.2 \ (-\,0.2)$
Total $\chi ^2$/dof	1386/1207	1430/1207	1479/1207

**Table 2:** The $\chi ^2$ values at NNLO for individual data sets for a selection of the charm matching scales $\mu _c$. The contribution of the charm data contained in the “Correlated $\chi ^2$” and in the “Log penalty $\chi ^2$” terms is indicated separately in the parentheses

Charm NNLO	$\mu _c = 1\, m_c$	$2\, m_c$	$3\, m_c$
Charm cross section H1-ZEUS combined	45/47	50/47	50/47
H1 F2 Beauty Vertex	3.5/12	3.5/12	3.3/12
Beauty cross section ZEUS Vertex	13/17	13/17	13/17
HERA1+2 CCep	43/39	43/39	43/39
HERA1+2 CCem	55/42	55/42	54/42
HERA1+2 NCem	217/159	217/159	217/159
HERA1+2 NCep 820	66/70	64/70	66/70
HERA1+2 NCep 920	444/377	433/377	442/377
HERA1+2 NCep 460	218/204	219/204	216/204
HERA1+2 NCep 575	220/254	218/254	219/254
Correlated $\chi ^2$ total (charm)	111 (10.8)	109 (11.3)	110 (14.5)
Log penalty $\chi ^2$ total (charm)	$+\,18\ (-\,1.1)$	$+\,18\ (-\,1.8)$	$+\,15\ (-\,1.8)$
Total $\chi ^2$/dof	1453/1207	1439/1207	1447/1207

The above smooth transition of $F_2^b(x,Q)$ from the $N_F=4$ to the $N_F=5$ scheme holds even though the PDFs and $\alpha _S(\mu )$ have discontinuities. Because we have used consistent choices for $\{ m_b, f_i^{(N_F)}, \alpha _S \}$, the cancellation of Sect. 2.3 applies, and the effect of any discontinuities in the physical observable will be of higher order. Conversely, a mismatch in $\{ m_b, f_i^{(N_F)}, \alpha _S \}$ would spoil this cancellation and result in unphysical large contributions when $f_b^{(5)}(x,\mu )$ is introduced. This is precisely the case where shifting the matching scale $\mu _b$ to a higher scale such as $2m_b$ or $3m_b$ would help avoid these problems.

It is interesting to note that, as we compute even higher orders, the discontinuities in the PDFs and $\alpha _S(\mu )$ will persist at lower order; but any discontinuities in the physical observables will systematically decrease order by order.

## The PDF fits

### xFitter, APFEL, and data sets 

To study the effects of varying the matching scales for the charm and bottom quark we will perform a series of fits to various data sets. Since we are varying the matching scales in the vicinity of $m_{c}$ and $m_{b}$, we want data that constrain the PDFs in this region. For this purpose, we include the very precise combined HERA data sets as these provide strong constraints in the region $\mu \sim m_{c,b}$, and they also extend up to higher scales [34–37]. In particular, the HERA measurement of the charm and bottom cross sections are included as they are sensitive to the choice of $\mu _c$ and $\mu _{b}$.

These fits are performed with the xFitter program using the APFEL evolution code [18, 38, 39]. The DIS calculations use the FONLL-B scheme for the NLO calculations, and the FONLL-C scheme for the NNLO calculations; these are both $\mathcal{O}(\alpha _S^2)$ prescriptions, and the details are specified in Ref. [6]. We use $m_c=1.45$ GeV, $m_b=4.5$ GeV, $\alpha _S(M_Z)=0.118$ for both the NLO and the NNLO calculations. The fit is performed using pole masses, but the formalism can be used equally well with the $\overline{\text{ MS }}$ definition of the heavy-quark masses [40]. For the PDFs, we use a HERAPDF 14-parameter functional form with initial QCD evolution scale $Q_0^2=1.0~\mathrm{GeV}^2$ and strangeness fraction $f_s=0.4$; the other QCD fit settings and constraints are similar to the analysis of Ref. [40].

The minimization of the $\chi ^2$ is performed using MINUIT [41]. The correlations between data points caused by systematic uncertainties are taken into account in the “Correlated $\chi ^2$” contribution. A “Log penalty $\chi ^2$” arises from the likelihood transition to $\chi ^2$ when the scaling of the errors is applied [16, 42].

The full sets of data are listed in Tables 1, 2, 3 and 4, and the reference for each data set is cited in Table 1. The combined inclusive HERA data (HERA1+2) from Ref. [34] includes both neutral current (NC) and charged current (CC) results for electrons (em) and positrons (ep) at a variety of energies. The charm cross sections from Ref. [36] include the combined H1-ZEUS results. The bottom cross sections from ZEUS are presented in Ref. [37] and those from H1 in Ref. [35].

**Table 3:** The $\chi ^2$ values at NLO for individual data sets for a selection of the bottom matching scales $\mu _b$. The contribution of the bottom data contained in the “Correlated $\chi ^2$” and in the “Log penalty $\chi ^2$” terms is indicated separately in the parentheses

Bottom NLO	$\mu _b = 1\, m_b$	$3\, m_b$	$5\, m_b$	$10\, m_b$	$14\, m_b$
Charm cross section H1-ZEUS combined	46/47	46/47	46/47	46/47	46/47
H1 F2 Beauty Vertex	3.1/12	3.2/12	3.1/12	3.2/12	3.2/12
Beauty cross section ZEUS Vertex	12/17	12/17	12/17	12/17	14/17
HERA1+2 CCep	44/39	44/39	44/39	44/39	44/39
HERA1+2 CCem	52/42	52/42	52/42	53/42	53/42
HERA1+2 NCem	220/159	219/159	220/159	219/159	219/159
HERA1+2 NCep 820	65/70	65/70	65/70	65/70	65/70
HERA1+2 NCep 920	414/377	410/377	410/377	412/377	412/377
HERA1+2 NCep 460	221/204	221/204	221/204	219/204	220/204
HERA1+2 NCep 575	216/254	216/254	216/254	216/254	216/254
Correlated $\chi ^2$ total (bottom)	86 (0.8)	86 (0.8)	86 (0.8)	87 (0.8)	89 (0.8)
Log penalty $\chi ^2$ total (bottom)	$+\,6.7\ (-\,0.1)$	$+\,4.2\ (-\,0.1)$	$+\,4.5\ (-\,0.1)$	$+\,6.6\ (-\,0.1)$	$+\,7.3\ (-\,0.1)$
Total $\chi ^2$/dof	1386/1207	1379/1207	1380/1207	1383/1207	1388/1207

**Table 4:** The $\chi ^2$ values at NNLO for individual data sets for a selection of the bottom matching scales $\mu _b$. The contribution of the bottom data contained in the “Correlated $\chi ^2$” and in the “Log penalty $\chi ^2$” terms is indicated separately in the parentheses

Bottom NNLO	$\mu _b = 1\, m_b$	$3\, m_b$	$5\, m_b$	$10\, m_b$	$14\, m_b$
Charm cross section H1-ZEUS combined	45/47	45/47	45/47	45/47	45/47
H1 F2 Beauty Vertex	3.5/12	3.7/12	3.7/12	3.6/12	3.6/12
Beauty cross section ZEUS Vertex	13/17	13/17	13/17	13/17	14/17
HERA1+2 CCep	43/39	43/39	43/39	42/39	42/39
HERA1+2 CCem	55/42	55/42	55/42	55/42	56/42
HERA1+2 NCem	217/159	216/159	220/159	218/159	218/159
HERA1+2 NCep 820	66/70	66/70	66/70	66/70	66/70
HERA1+2 NCep 920	444/377	445/377	445/377	451/377	453/377
HERA1+2 NCep 460	218/204	219/204	219/204	217/204	218/204
HERA1+2 NCep 575	220/254	219/254	219/254	219/254	219/254
Correlated $\chi ^2$ total (bottom)	111 (0.9)	112 (0.9)	112 (0.9)	114 (0.9)	116 (0.9)
Log penalty $\chi ^2$	+ 18	+ 17	+ 15	+ 18	+ 18
Total $\chi ^2$/dof	1453/1207	1453/1207	1457/1207	1463/1207	1470/1207

### Impact of matching on the fits: charm

**Fig. 5:**
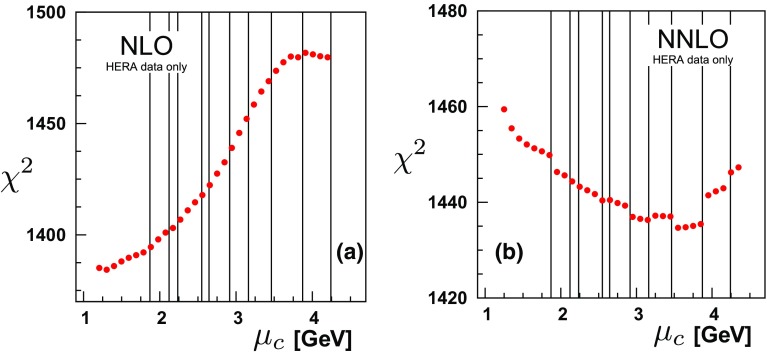
$\chi ^{2}$ vs. the charm matching scale $\mu _{c}$ at **a** NLO and **b** NNLO for all data sets. The bin boundaries for the HERA data set “HERA1+2 NCep 920” are indicated by the vertical lines

**Fig. 6:**
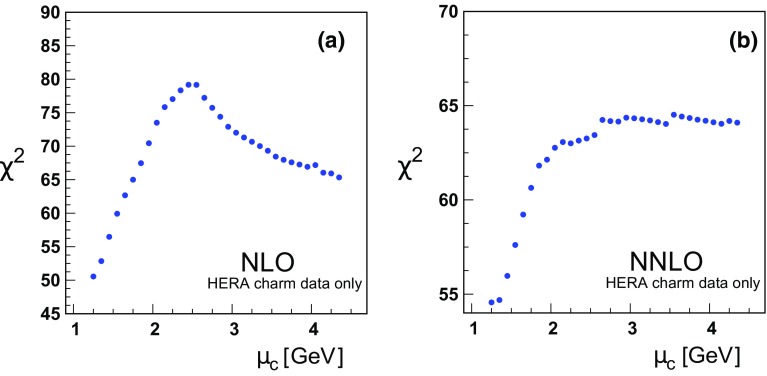
$\chi ^{2}$ vs. the charm matching scale $\mu _{c}$ at **a** NLO and **b** NNLO for only the H1-ZEUS combined charm production data; note, this includes the correlated $\chi ^2$ contribution from Tables 1 and 2

The charm cross section data are expected to be sensitive to the treatment of the charm PDF in the threshold region, and this is reflected in the results of Figs. 5, 6 and Tables 1, 2.

Figure 5 displays the results for varying the charm quark matching scale $\mu _{c}$ both for the NLO and NNLO calculations.^9^ Comparing the NLO and NNLO cases, the NLO result ranges over $\sim 100$ units in $\chi ^{2}$, while the NNLO varies over $\sim 25$ units of $\chi ^{2}$. This difference in the $\chi ^2$ variation reflects the effects of the higher-order terms; it is reassuring to see that the $\mu _c$ dependence decreases at higher orders.

At NLO, the matching conditions pick up the contribution of only the single log term *L* (Eq. (6)), while at NNLO we pick up both the *L* and the $L^2$ terms. In contrast, the DGLAP evolved charm PDF resums the above, as well as an infinite tower of logs: $\sum _{n=1}^{\infty } \sum _{k=0}^{n} \alpha _S^n \, L^k$.

Examining the NLO analysis of Fig. 5a, we find that at low scales, the $\chi ^2$ increases with increasing $\mu _c$ scale. While our plot extends slightly below the charm mass, it is not obvious if there is actually a minimum in $\mu _c$. It is problematic to compute with $\mu _c$ values much lower than $m_c$ as $\alpha _S$ becomes large and the charm PDF negative. Thus, the optimal computational range for $\mu _c$ appears to be in the region of $m_c$.

Focusing on the charm data alone as shown in Fig. 6a, the situation is not so clear; the $\chi ^2$ increases with increasing $\mu _c$, but again there does not appear to be a minimum at low $\mu _c$ values. Moving to large $\mu _c$, the $\chi ^2$ values initially increase, but then decrease as $\mu _c$ approaches $m_b$. As we want to maintain the ordering $\mu _c<\mu _b$, we cannot go to larger scales unless we increase $\mu _b$. While this is allowed, it is more complex to explore the two-dimensional $\{ \mu _c, \mu _b \}$ parameter space; hence, we limit the present study to variation of a single scale.

The $\chi ^2$ results for each individual data set is summarized in Table 1. The data sets with the largest effects are (i) the H1-ZEUS combined charm cross section data, and (ii) the very precise “HERA1+2NCep 920” set. The sensitivity of the “HERA1+2NCep 920” set is due to a large number of data points with small uncertainties.

Turning to the NNLO analysis of Fig. 5b and the results of Table 2, a number of points are evident. Again, the two data sets with the largest impact are the H1-ZEUS combined charm cross section data, and the “HERA1+2NCep 920” set. In Fig. 5 the vertical lines indicate the bin boundaries for the “HERA1+2NCep 920” data set.

Scanning in $\chi ^2$, discrete jumps are evident. As we vary the matching scale, certain data bins move between the $N_{F}=3$ and $N_{F}=4$ schemes, shifting the $\chi ^2$ by one or two units which is visible in Fig. 5b. These jumps reflect the underlying theoretical uncertainty arising from the choice of $N_F$.

In Fig. 5b the total NNLO variation of $\chi ^2$ is reduced compared to the NLO case, and the minimum global $\chi ^2$ is now in the region $\mu _c \sim 2 m_c$. Focusing on the charm data alone in Fig. 6b, again it is not obvious if there is actually a minimum in $\mu _c$. Given the limitations of computing with $\mu _c \ll m_c$, the optimal computational range again appears to be in the general region of $m_c$.

While it may be tempting to try and optimize the matching scale for each data set, recall that $\mu _{m}$ represents a choice of scheme, and thus reflects an inherent theoretical uncertainty; a specific choice of $\mu _{m}$ will not reduce this uncertainty.

This situation can also be found in complex global fits where the final result may be a compromise of data sets which are in tension; this is why a tolerance factor is often introduced. This complexity is evident when examining the details of Tables 1 and 2 which demonstrate the minimum $\chi ^2$ for individual data sets is not simply correlated; this will be discussed further in Sect. 4.4. An additional challenge of analyzing the charm case is that $\mu _c$ can only vary over the limited dynamic range between $\sim m_c$ and $\mu _b$. This will not be an issue for the bottom quark (because $m_t \gg m_b$), which is considered in the following section.

### Impact of matching on the fits: bottom

**Fig. 7:**
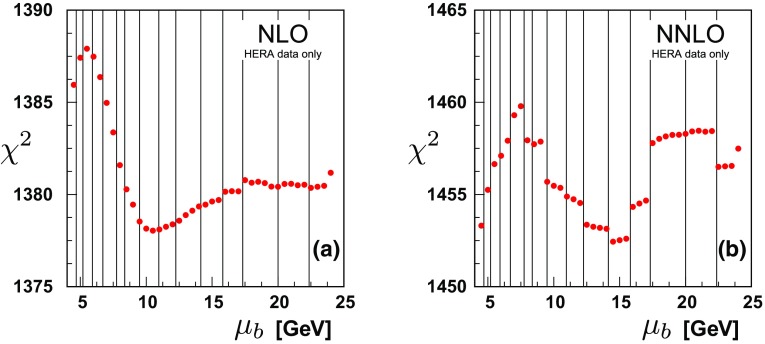
$\chi ^{2}$ vs. the bottom matching scale $\mu _{b}$ at **a** NLO and **b** NNLO for all data sets. The bin boundaries for the HERA data set “HERA1+2 NCep 920” are indicated by the vertical lines

Figure 7 presents the results for varying the bottom-quark matching scale $\mu _{b}$ both for the NLO and NNLO calculations. This figure highlights the ranges of $\chi ^2$; the NLO result ranges over approximately $\sim 10$ units in $\chi ^{2}$, and the NNLO varies by about the same amount.

**Fig. 8:**
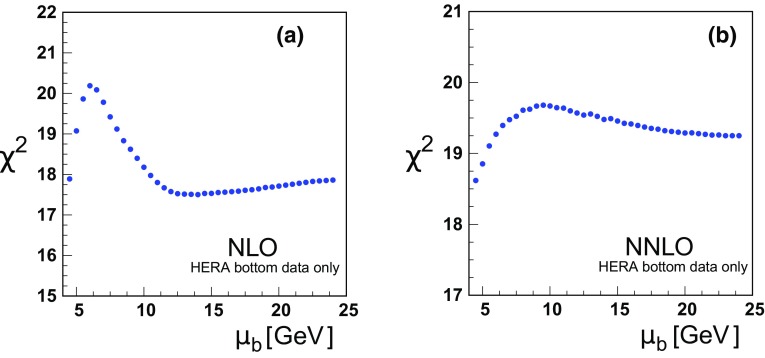
$\chi ^{2}$ vs. the bottom matching scale $\mu _{b}$ at **a** NLO and **b** NNLO for only the bottom data; note, this includes the H1 and ZEUS beauty data as well as the correlated $\chi ^2$ contribution from Tables 3 and 4

The reduced $\chi ^{2}$ variation as compared to the charm case reflects, in part, the decrease in the strong coupling $\alpha _{S}(m_{b})<\alpha _{S}(m_{c})$, which also diminishes the higher-order contributions. Figures 5 with 7 there is a $\chi ^{2}$ range of $\sim 100$ vs. $\sim 10$ for NLO, and $\sim 15$ vs. $\sim 10$ for NNLO.

Examining the NLO analysis of Fig. 7a, there is a slight minimum for $\chi ^2$ in the region $\mu _b \sim 2 m_b$ with relatively flat behavior at larger $\mu _b$ scales. Correspondingly, there is a similar behavior when we focus on only the bottom data of Fig. 8a. The $\chi ^2$ results for each individual data set is summarized in Table 3.

The data sets with the largest effects are (i) the very precise “HERA1+2NCep 920” set, and (ii) the separate H1 and ZEUS bottom cross section data. The H1 and ZEUS bottom cross sections display some minimal $\chi ^2$ variation in the region $\mu _b \sim m_b$, but then is relatively flat out to very high scales ($\mu _b \sim 14 m_b$). It is primarily the “HERA1+2NCep 920” set which drives the shape of the $\chi ^2$ curve in the $\mu _b\sim m_b$ region. Compared to the charm results, the interpretation of the bottom cross section data requires some care as the number of data points is smaller, and the relative uncertainty larger.

Turning to the NNLO analysis of Fig. 7b, the variation of the $\chi ^2$ curve is within $\sim 8$ units across the range of the plot. The resolution of the vertical $\chi ^2$ scale accentuates the discrete jumps as the data bins move between the $N_{F}=4$ and $N_{F}=5$ schemes. The bin boundaries for the “HERA1+2NCep 920” data set are indicated with vertical lines.

Focusing on the bottom data alone as shown in Fig. 8b, the $\chi ^2$ profile is flat within one unit across the plot range.

For both Figs. 7b and 8b, the $\chi ^2$ variation is within a reasonable “tolerance” factor for the global fit; thus, the matching scale $\mu _b$ can vary within this range with minimal impact on the resulting fit.

The scale $\mu _b$ can extend up to larger scales, and Tables 3 and 4 display the results for $10m_b$ and $14m_b$. The pattern across the various data sets is consistent, and the overall $\chi ^2$ values rise slowly.

### Comparisons

**Fig. 9:**
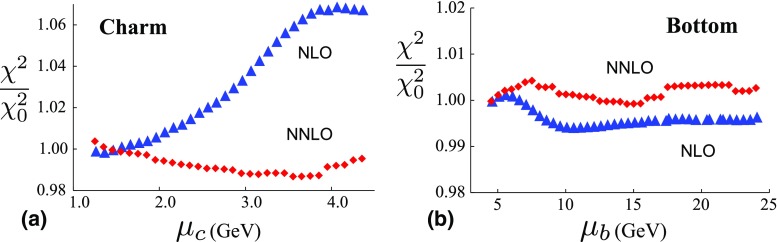
The ratio ($\chi ^2/\chi ^2_0$) of total $\chi ^2$ values (all data sets combined) from Figs. 5 and 7, as a function of the **a** charm and **b** bottom matching scale $\mu _{c,b}$ in GeV. $\chi _{0}^{2}$ is the $\chi ^{2}$ value for $\mu _{m}$ equal to the quark mass. The triangles (blue filled triangle) are NLO and the diamonds (red filled lozenge) are NNLO

**Fig. 10:**
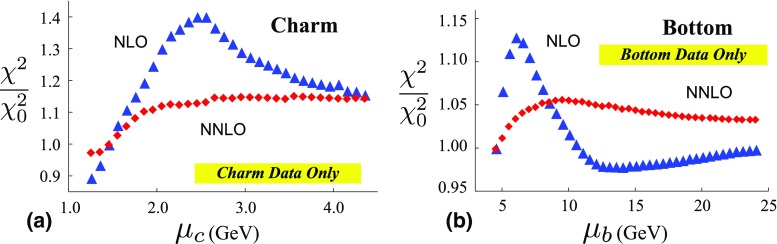
The ratio ($\chi ^2/\chi ^2_0$) of partial $\chi ^2$ values (charm/bottom data only) from Figs. 6 and 8 as a function of the **a** charm and **b** bottom matching scale $\mu _{c,b}$ in GeV. $\chi _{0}^{2}$ is the $\chi ^{2}$ value for $\mu _{m}$ equal to the quark mass. The triangles (blue filled triangle) are NLO and the diamonds (red filled lozenge) are NNLO

To facilitate comparisons of the NLO and NNLO results, Fig. 9 displays the ratio $\chi ^{2}/\chi ^{2}_0$ for charm (on the left) and bottom (on the right) where $\chi ^{2}_0$ is the value of the $\chi ^2$ at $\mu _m=m_H$. Similarly, Fig. 10 displays the same ratio for only the heavy-quark data sets. By plotting $\chi ^{2}/\chi ^{2}_0$, we can better compare the fractional variation of $\chi ^2$ across the matching scale values.

The motivation for the scaled plot of Figs. 9 and 10 is that the overall $\chi ^2$ values are different; specifically, those of the NNLO are greater than the NLO. This counter intuitive result has been observed in other analyses [34, 43], and it has been suggested that this may be improved by resumming the singular $\ln [1/x]$ terms in the higher-order splitting kernels [44].

Here, we first make some observations specific to Figs. 9 and 10.

- At NLO for the case of charm, the optimal computational scale for $\mu _c$ is in the general range $\mu _c\sim m_c$ for both the inclusive data set (Fig. 9a) and the charm data set (Fig. 10a). For lower scales ($\mu _c \ll m_c$), $\alpha _S(\mu )$ is large and the charm PDFs are negative. For higher scales ($\mu _c \gg m_c$), $\chi ^2/\chi ^2_0$ increases.
- At NLO for the case of bottom, the optimal scale for $\mu _b$ is in the general range $\mu _b\sim 2 m_b$. For the inclusive data set (Fig. 9b) the $\chi ^2/\chi ^2_0$ variation is very mild ($\sim 1\%$), while for the bottom data set (Fig. 10b) the $\chi ^2/\chi ^2_0$ variation is larger ($\sim 10\%$).
- At NNLO for the case of charm, the $\chi ^2/\chi ^2_0$ variation is reduced. For the inclusive data set (Fig. 9a) the $\chi ^2/\chi ^2_0$ variation is very mild ($\sim 2\%$), while for the charm data set (Fig. 10a) the $\chi ^2/\chi ^2_0$ variation is larger ($\sim 10\%$). There is no obvious optimal choice for the $\mu _c$ scale.
- At NNLO for the case of bottom, the $\chi ^2/\chi ^2_0$ variation is reduced and a matching scale choice in the region $\mu _b\sim m_b$ appears to be optimal. For the inclusive data set (Fig. 9b) the $\chi ^2/\chi ^2_0$ variation is very mild ($\sim 1\%$), while for the bottom data set (Fig. 10b) the $\chi ^2/\chi ^2_0$ variation is slightly larger ($\sim 5\%$).

While the detailed characteristics of the above fits will depend on specifics of the analysis, there are two general patterns which emerge: (i) the $\chi ^2$ variation of the NNLO results are generally reduced compared to the NLO results, and (ii) the relative $\chi ^2$ variation across the bottom transition is reduced compared to the charm transition. For example, although the global $\chi ^2$ can be modified by different choices of data sets and weight factors, these general properties persist for each individual data set of Tables 1, 2, 3 and 4; in fact, we see that the bulk of the data sets are quite insensitive to the details of the heavy-quark matching scale. Additionally, there are a variety of prescriptions for computing the heavy flavor contributions; these primarily differ in how the higher-order contributions are organized. As a cross check, we performed a NLO fit using the FONNL-A scheme; while the absolute value of $\chi ^2$ differed, the above general properties persisted.

The net result is that we can now quantify the theoretical uncertainty associated with the transition between different $N_F$ sub-schemes. In practical applications, if we choose $\mu _{c}\sim m_c$, the impact of the $N_F=3$ to $N_F=4$ transition is reduced as this is often below the minimum kinematic cuts of the analysis (e.g. $Q_{min}^2$ and $W_{min}^2$). Conversely, the $N_F=4$ to $N_F=5$ transition is more likely to fall in the region of fitted data; hence, it is useful to quantify the uncertainty associated with the $\mu _b$ choice.

## An example: $N_F$-dependent PDFs

The variable matching scale $\mu _m$ can be used as an incisive tool to explore various aspects of the PDFs and global fits. As an example, Ref. [22] introduced an $N_F$-dependent PDF $f_{i}(x,\mu ,N_{F})$ where $N_F$ is the active number of flavors in the VFNS. This extension provides additional flexibility in the region of the heavy-quark thresholds; however, the implementation of Ref. [22] only used a fixed matching scale of $\mu _m = m_H$. Using xFitter we can improve on this concept by generating PDFs with a variable $\mu _m$ scale. We illustrate this below and provide example grids at xFitter.org.

**Fig. 11:**
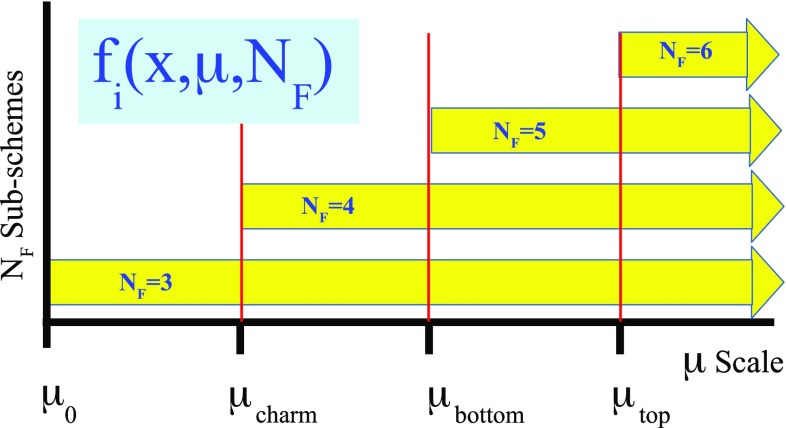
An illustration of the separate $N_{F}$ renormalization sub-schemes which define the VFNS. In contrast to Fig. 1a, each of the $N_{F}$ sub-schemes are available for all scales above $\mu _{m}$. The particular scheme can be specified by choosing $N_{F}$ when calling the PDF, i.e. $f_{i}(x,\mu ,N_{F})$. This illustration shows a matching scale of $\mu _{m}=m_H$

**Fig. 12:**
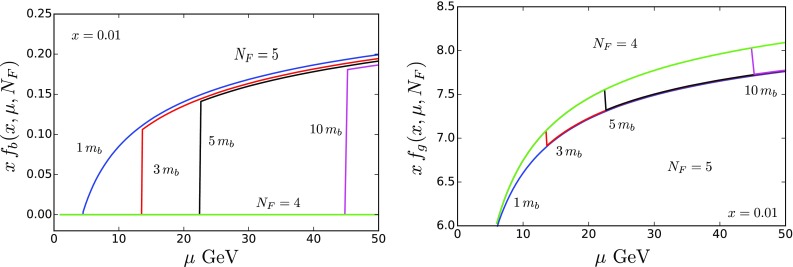
$N_F$-dependent PDFs $x \, f_i(x,\mu ,N_F)$ for the bottom quark (left) and gluon (right) with variable matching scales for $\mu _b=\{1,3,5,10, \infty \}\times m_b$ {blue, red, black, magenta, green} with $x=0.01$ as a function of $\mu$ in GeV. The vertical lines in the plots show the transition from the $N_F=4$ to $N_F=5$ flavor scheme

The usual PDF can be generalized to include an $N_F$-dependence [22]: $f_{i}(x,\mu )\rightarrow f_{i}(x,\mu ,N_{F})$. In this approach, the many $N_{F}=\{3,4,5,\ldots \}$ flavor schemes coexist, and they can be selected by specifying the number of active flavors $N_F$ along with the other arguments of the PDF. This concept is represented pictorially in Fig. 11. All the $N_{F}$ sets of PDFs are available above the matching scale $\mu _{m}$. For example, with an $N_F$-dependent PDF, one could simultaneously analyze selected data sets with $N_F=4$ and others with $N_F=5$ even if they overlap kinematically; the user has the flexibility (and responsibility) to select $N_F$.

Note in Fig. 11 that the various $N_F$ grids are not individual fits but are related analytically via the flavor threshold matching conditions. Operationally, they are generated from an initial PDF $f_i(x,\mu _0,N_F=3)$ and $\alpha _S(\mu _0)$ at the starting scale $\mu _0$. The $N_F=3$ grid is generated by evolving from $\mu _0$ to $\mu _{\mathrm{max}}$. The $N_F=4$ grid is then generated by matching at $\mu _c$ (which may or may not equal $m_c$), and evolving up to scale $\mu _{\mathrm{max}}$. The $N_F=5$ and $N_F=6$ grids are generated in a similar manner.^10^ This process ensures that all the PDFs $f_i(x,\mu ,N_F)$ are analytically related to the PDF and $\alpha _S$ boundary conditions at $\mu _0$.

To provide an explicit illustration of the above, we have generated a set of PDF grids with a variety of matching scales ($\mu _b$) for the matching between the schemes with $N_F=4$ and $N_F=5$ active flavors: $\mu _{b}=\{1,3,5,10, \infty \}\times m_b$. We focus on $\mu _{b}$ as this is the flavor transition most likely to fall within a particular data set. For the initial PDF we use the NNLO bottom fit with $\mu _b=1\, m_b$ of Table 4, and we evolve at NNLO. The PDFs are fixed such that they all match at the initial evolution scale $\mu _0=1.0$ GeV with the same value of $\alpha _S(\mu _0)= 0.467464$.

This is illustrated in Fig. 12 where we display the bottom quark and gluon PDFs as a function of $\mu$ in GeV. As we evolve up in $\mu$, we explicitly see the transition from $N_F=4$ to $N_F=5$ flavors at each respective $\mu _b$ threshold. For these particular kinematic values, the discontinuity of the bottom PDF is positive while that of the gluon is negative; this ensures the momentum sum rule is satisfied. Furthermore, we observe the spread in the bottom PDF at large $\mu$ is broader than that of Fig. 3. In Fig. 12, while the values of $\alpha _S$ all coincide at $\mu _0$, the evolution across the different $\mu _b$ thresholds result in different $\alpha _S$ values at large $\mu$ scales. This is in contrast to Fig. 3 where the values of $\alpha _S$ all coincide at the large scale $\mu =M_Z$. Additionally, note that the illustration in Fig. 3 is based on the NNPDF3.0 PDF set while Fig. 12 is based on our fit from Table 4.

Because the $N_F=4$ and $N_F=5$ grids are available concurrently, we can choose to analyze the HERA data in an $N_F=4$ flavor scheme for arbitrarily large scales, but simultaneously allow LHC data to be analyzed in a $N_F=5$ flavor scheme throughout the full kinematic region even down to low scales.

In this illustration, the PDFs revert to $N_F=4$ below $\mu _b$; however, this is not required. For example the $N_F=5$ PDFs could be evolved backwards from $\mu _b$ to provide values at scales $\mu <\mu _b$. Both APFEL [18] and QCDNUM [46, 47] have this capability.^11^

For bottom at NNLO using the results from Table 4 for the inclusive data set, we observe the $\mu _b$ variation is minimal. Thus, a choice in the range $\mu _{b}\sim [m_{b}, 5 m_b]$ yields a $\varDelta \chi ^2 \le (1457-1453)\sim 4$ units out of $\sim 1450$. This minimal $\chi ^2$ dependence means we can shift the $\mu _b$ matching scale if, for example, we want to avoid a $N_F$ flavor transition in a specific kinematic region. While these results should be checked with additional data sets, the insensitivity to $\mu _{b}$, especially at NNLO, is an important result as the ability to displace the $N_{F}=4$ and $N_{F}=5$ transition can be beneficial when this threshold comes in the middle of a data set.

Combined with the variable heavy-quark threshold, the $N_F$ dependent PDFs provide additional flexibility to analyze multiple data sets in the optimal theoretical context.

## Conclusions

In this study we have examined the impact of the heavy flavor matching scales $\mu _m$ on a PDF fit to the combined HERA data set.

The choice of $\mu _m$ allows us to avoid delicate cancellations in the region $\mu _m\sim m_H$ as illustrated in Fig. 2. Additionally, the discontinuities associated with the $N_F=4$ to $N_F=5$ transition can be shifted so that these discontinuities do not lie in the middle of a specific data set.

Using xFitter and APFEL to study the $\mu _m$ dependence of a global PDF fit to the HERA data, we can extract the following general features. For the charm matching scale, $\mu _c$, there is a large variation of $\chi ^{2}$ at NLO, but this is significantly reduced at NNLO. In contrast, for the bottom matching scale, $\mu _b$, there is a relatively small variation of $\chi ^{2}$ at both NLO and NNLO.

These observations can be useful when performing fits. While charm has a larger $\chi ^{2}$ variation (especially at NLO), the charm quark mass $m_c\sim 1.45$ GeV lies in a region which is generally excluded by cuts in $Q^2$ and/or $W^2$.

On the contrary, the $\chi ^{2}$ variation for the bottom quark is relatively small at both NLO and NNLO. Since the bottom-quark mass $m_b\sim 4.5$ GeV is in a region where there is abundance of precision HERA data, this flexibility allows us to shift the heavy flavor threshold (and the requisite discontinuities) away from any particular data set. Functionally, this means that we can analyze the HERA data using an $N_F=4$ flavor scheme up to relatively large $\mu$ scales, and then perform the appropriate NNLO matching (with the associated constants and log terms) so that we can analyze the high-scale LHC data in the $N_F=5$ or even $N_F=6$ scheme.

These variable heavy flavor matching scales $\mu _m$ allow us to generalize the transition between a FFNS and a VFNS, and provides a theoretical “laboratory” which can quantitatively test proposed implementations. We demonstrated this with the example of the $N_F$-dependent PDFs. Having the quantitative results for the $\chi ^2$ variation of the $\mu _{c,b}$ scales, one could systematically evaluate the impact of using different matching scale choices for the $f_i(x,\mu ,N_F)$.

In conclusion, we find that the ability to vary the heavy flavor matching scales $\mu _m$, not only provides new insights into the intricacies of QCD, but also has practical advantages for PDF fits.

## Footnotes

1 The perturbative coefficients of $\mathcal{M}_{i}^{j}$ at NLO are available in Refs. [12, 13], and at NNLO in Ref. [14]. $m_H$ is the mass of the $N_F+1$ flavor quark. For $\alpha _S(\mu )$, the $c_{n \, k}$ coefficients are available in the Particle Data Group review of Quantum Chromodynamics [15].

2 Information on the xFitter program can be found at http://www.xFitter.org, and in Refs. [16, 17].

3 For the CWZ scheme with $N_F$ (active) flavors and an arbitrary number of heavy (inactive) flavors, the evolution of the PDFs and $\alpha _S^{(N_F)}(\mu )$ will involve only the active $N_F$ flavors; the inactive heavy flavors can be ignored.

4 In Eq. (4), $\widetilde{f}_b(x,\mu )$ includes the single splitting $(g\rightarrow b \bar{b})$; in contrast, the DGLAP evolution of $f_b(x,\mu )$ sums an infinite tower of splittings. Note, we have used the NNPDF30_lo_as_118_nf_6 PDFs to precisely match the order of the splitting kernels in the NLO calculation.

5 The above correspondences are only approximate as the VFNS and FFNS also differ in $\alpha _S$ and the PDFs.

6 A first study of the impact of moving the bottom matching scale with respect to the bottom mass was already done in Ref. [28] in the context of $\overline{b}bH$ production at the LHC using a matched scheme. The approach developed in this study was more recently applied to the 13 TeV LHC in Ref. [29].

7 At NNLO, the bottom-quark matching condition also receives contributions from the light quarks as well as gluons; this has been included in the calculation.

8 While the VFNS framework is compatible with an intrinsic charm or bottom PDF, we do not introduce these into the current study. For additional details, see Refs. [30–33].

9 For these scans we hold the bottom matching fixed at $\mu _{b}=m_{b}$ and keep $\mu _{c}<m_{b}$ so the ordering of the mass thresholds is not inverted.

10 Note the $N_F=\{3,4,5,6\}$ grids are stored in separate LHAPDF data files; they can be combined into an effective $N_F$ dependent PDF as illustrated in Refs. [22, 45].

11 However, it is generally advisable not to backwards evolve too far in $\mu$ as this can become unstable [48, 49].
